# Epigenetic silencing of miR‐1271 enhances MEK1 and TEAD4 expression in gastric cancer

**DOI:** 10.1002/cam4.1605

**Published:** 2018-06-04

**Authors:** Byungho Lim, Hee‐Jin Kim, Haejeong Heo, Nanhyung Huh, Su‐Jin Baek, Jong‐Hwan Kim, Dong‐Hyuck Bae, Eun‐Hye Seo, Sang‐Il Lee, Kyu‐Sang Song, Seon‐Young Kim, Yong Sung Kim, Mirang Kim

**Affiliations:** ^1^ Division of Drug Discovery Research Research Center for Drug Discovery Technology Korea Research Institute of Chemical Technology Daejeon Korea; ^2^ Genome Editing Research Center Korea Research Institute of Bioscience and Biotechnology (KRIBB) Daejeon Korea; ^3^ Personalized Genomic Medicine Research Center Korea Research Institute of Bioscience and Biotechnology (KRIBB) Daejeon Korea; ^4^ Department of Functional Genomics University of Science and Technology (UST) Daejeon Korea; ^5^ Department of Surgery College of Medicine Chungnam National University Daejeon Korea; ^6^ Department of Pathology College of Medicine Chungnam National University Daejeon Korea

**Keywords:** DNA methylation, gastric cancer, MEK1, miR‐1271, TEAD4

## Abstract

Epigenetic dysregulation is a major driver of tumorigenesis. To identify tumor‐suppressive microRNAs repressed by DNA methylation in gastric cancer (GC), we analyzed the genome‐wide DNA methylation and microRNA expression profiles of EpCAM+/CD44+ GC cells. Among the set of microRNAs screened, miR‐1271 was identified as a microRNA repressed by DNA methylation in GC. Forced miR‐1271 expression substantially suppressed the growth, migration, and invasion of GC cells. To identify candidate target genes and signaling pathways regulated by miR‐1271, we performed RNA sequencing. Among the genes down‐regulated by miR‐1271, *MAP2K1* (MEK1) was significantly repressed by miR‐1271, and the associated ERK/MAPK signaling pathway was also inhibited. TEAD4 was also repressed by miR‐1271, and the associated YAP1 signatures within genes regulated by miR‐1271 were significantly enriched. These findings uncovered MEK1 and TEAD4 as novel miR‐1271 targets and suggest that the epigenetic silencing of miR‐1271 is crucial for GC development.

## 1 INTRODUCTION

Epigenetic mechanisms, including DNA methylation, histone modifications, and chromatin remodeling, play pivotal roles in the development, differentiation, and maintenance of cellular systems by controlling gene expression. Thus, dysregulation of epigenetic mechanisms induces pathogenic outcomes, including cancer. DNA methylation has been extensively investigated and found to be altered during tumorigenesis, thereby resulting in cancer type‐specific DNA methylation signatures.^1^, ^2^, ^3^ Accordingly, DNA methylation has important implications in the molecular classifications of multiple cancer types.^4^ Additionally, aberrant DNA methylation, associated with overexpression of oncogenes via DNA demethylation and down‐regulation of tumor suppressors via DNA hypermethylation, is involved in all steps of tumorigenesis, including cancer initiation, progression, and metastasis.^5^ Therefore, a comprehensive investigation of DNA methylation patterns provides an opportunity to identify important drivers of cancer.

MicroRNAs (miRNAs) are a class of small noncoding RNAs that are ~22 nucleotides in length.^6^ In addition to other epigenetic mechanisms, miRNAs act as an additional epigenetic regulator by posttranscriptionally repressing target mRNAs by binding their 3′ untranslated regions (UTRs). To date, over 1900 distinct miRNAs have been discovered throughout the human genome,^7^ potentially regulating at least 30% of all protein‐coding genes.^8^ The large numbers of miRNAs suggests the importance of their roles in a wide array of biological processes, including several diseases. Numerous miRNAs have been found to be involved in the pathogenesis of multiple types of cancer. Additionally, miRNA expression itself is dysregulated in human malignancies via several mechanisms, including amplification or deletion of genomic regions containing miRNAs, abnormal transcriptional regulation of miRNAs, and defective epigenetic changes.^9^

In this study, we attempted to analyze the methylome and miRNome of gastric cancer (GC) cells on a genome‐wide level. To identify tumor‐suppressive miRNAs repressed by DNA hypermethylation in highly tumorigenic GC, we isolated EpCAM+/CD44+ GC cells from primary GC tumor tissues and performed methyl‐CpG‐binding domain (MBD) sequencing (MBD‐seq) and miRNA sequencing (miRNA‐seq). Among a set of hypermethylated and down‐regulated miRNAs, we especially focused on miR‐1271 and demonstrated its tumor‐suppressive function in GC. Additionally, we further identified MEK1 and TEAD4 as miR‐1271 targets.

## 2 MATERIALS AND METHODS

### 2.1 Tissue samples and cell lines

To profile the methylome and miRNome of GC, 3 GC and adjacent normal tissue samples were obtained, along with informed consent, from Pusan National University Yangsan Hospital in Korea. EpCAM+/CD44+ GC cells were isolated from the 3 GC tissues according to a previously described protocol.^10^ To measure the protein expression levels of miR‐1271 target genes in GC tissues, 3 paired normal tissues and GC tissues were obtained, along with informed consent, from Chungnam National University Hospital in Korea. These studies were approved by the Internal Review Board at the corresponding hospitals, and all the experiments were performed in accordance with the relevant guidelines and regulations.

Nine GC cell lines (SNU‐1, SNU‐216, SNU‐484, SNU‐601, SNU‐620, AGS, KATO III, MKN1, and MKN74) and 293T cells were purchased from Korean Cell Line Bank (Seoul, Korea). The GC cell lines and 293T cells were cultured in Roswell Park Memorial Institute (RPMI) 1640 medium and Dulbecco’s Modified Eagle’s Medium (DMEM, WELGENE, Daegu, Korea), respectively, supplemented with 10% fetal bovine serum (FBS) (HyClone, Logan, UT, USA) in a CO_2_ incubator, and the cell lines were authenticated by short tandem repeat (STR) DNA profile analysis performed by the Korean Cell Line Bank facility.

### 2.2 MBD‐seq and data analysis

MBD‐seq was performed using EpCAM+/CD44+ GC cells and GC cell lines according to a previously reported method.^11^ Briefly, MBD2‐immunoprecipitated chromatin fragments were collected, and genomic libraries were constructed using a TruSeq ChIP Sample Prep Kit (Illumina, San Diego, CA, USA). After sequencing the libraries using an Illumina Hiseq‐2000 sequencing system, 76‐bp single‐end reads were aligned to human reference genome 19 (hg19) using Burrows‐Wheelers Aligner (BWA)^12^ by executing a “bwa mem” command, and duplicates were removed by the Picard “MarkDuplicates” function (Broad Institute, Cambridge, MA, USA). The MEDIPS R package (v. 1.18.0) was used to identify differentially methylated regions from MBD‐seq data by executing the “MEDIPS.meth” and “MEDIPS.selectSig” commands. Homer (v.4.7) software was used to find and annotate methylation peaks by executing the “findPeaks” command.

### 2.3 miRNA‐seq, RNA‐seq, and data analysis

miRNA‐seq and data analysis were performed according to a previously reported method.^13^ In brief, RNAs were isolated using a mirVana miRNA Isolation Kit (Thermo Fisher Scientific, Waltham, MA, USA), and libraries were prepared using an Illumina TruSeq Small RNA Sample Prep Kit. After sequencing on the Illumina HiSeq‐2000 system, adaptor sequences were removed using Trimmomatic,^14^ and the resulting reads were aligned to hg19 using TopHat v2.0.6.^15^ RNA‐seq and data analysis were performed according to a previously reported method.^16^ Briefly, total RNA was isolated using an RNeasy Mini Kit (Qiagen, Venlo, Netherlands), and libraries were prepared using an Illumina TruSeq RNA Sample Prep Kit v2. After sequencing on the Illumina HiSeq‐2000 sequencer, the resulting reads were aligned to hg19 using TopHat v2.0.6. To estimate the expression levels of the transcripts, we calculated the reads per kilobase per million mapped reads (RPKM) using custom Python scripts, which computed the mapped reads/(gene length/1000 × total number of reads/1 000 000). Using a list of genes ranked by fold change after miR‐1271 transfection, GSEA (GSEAPreranked) was performed to find miRNA motifs (c3.mir.v5.2.symbols.gmt from MSigDB) enriched within 3′UTR regions and cancer‐associated signaling pathways (c6.all.v5.2.symbols.gmt from MSigDB).^17^

### 2.4 miRNA expression

A synthetic mimic and miR‐1271 inhibitor were purchased from Thermo Fisher Scientific. Cells (2 × 10^5^) cultured in a 6‐well plate (SPL, Pocheon, Korea) were transfected with 100 pmoles of miRNA using Lipofectamine RNAiMAX (Invitrogen, Carlsbad, CA, USA). To establish miR‐1271‐expressing stable cell lines, 293T cells were cotransfected with MISSION Lentiviral Packaging Mix (Sigma‐Aldrich, St. Louis, MO, USA) and either a nonsilencing control vector, a miR‐1271 vector, or a miR‐1271 inhibitor vector (Applied Biological Materials Inc., Richmond, BC, USA). Over 3 days, supernatants containing lentivirus were collected from the 293T cells, filtered, and applied to target cells for lentiviral transduction. After 10 hours, the medium was changed to complete RPMI medium. After 1‐2 weeks of puromycin selection (Invitrogen), miR‐1271 expression was confirmed using qRT‐PCR.

### 2.5 Real‐time quantitative RT‐PCR (qRT‐PCR)

For mRNA expression analysis, qRT‐PCR was performed according to a previously reported protocol.^18^ Briefly, total RNA was isolated using an RNeasy Mini Kit (Qiagen), and cDNA was created using an iScriptTM cDNA Synthesis Kit (Bio‐Rad, Hercules, CA, USA). PCR reactions were prepared with iQTM SYBR Green Supermix and performed using a C1000TM Thermal Cycler (Bio‐Rad). The gene encoding β‐actin was amplified as a control, and relative quantifications of target mRNAs were analyzed using the comparative threshold cycle (CT) method.^19^ PCR primer sequences are listed in Table S2.

For miRNA expression analysis, qRT‐PCR was performed according to a previously reported protocol.^20^ Total RNA was purified using a mirVana miRNA Isolation Kit (Thermo Fisher Scientific) and reverse transcribed using specific primers and a TaqMan MicroRNA Reverse Transcription Kit (Applied Biosystems, Foster city, CA, USA). The resulting cDNAs were amplified and detected using real‐time PCR with specific stem‐loop primers and TaqMan probes from TaqMan MicroRNA Assays (Applied Biosystems). RNU6B snRNA (Applied Biosystems) was used as an internal control.

### 2.6 Bisulfite sequencing

Genomic DNA (2 μg) from each sample was modified by sodium bisulfite using the EZ DNA methylation kit (Zymo Research, Orange, CA, USA), according to the manufacturer’s instructions and PCR amplified. The PCR products (568 bp) were cloned into pGEM‐T Easy vector (Promega), and several clones were randomly chosen for sequencing. Bisulfite‐modified DNA was amplified using primer sets designed to amplify CpG133 region for miR‐1271 using MethPrimer (http://www.urogene.org/cgi-bin/methprimer/methprimer.cgi). Primer sequences used for miR‐1271 are 5′‐GAGTTAATTTTTGGTGGATGTTAGTAAGTA‐3′ (forward) and 5′‐TAATCACACCCCTTAACCACATAC‐3′ (reverse).

### 2.7 5‐Aza‐2′‐deoxycytidine treatment

GC cells (SNU‐601 and MKN74) were seeded on 100‐mm dishes at 3 × 10^5^ cells/dish and treated with 10 μmol/L 5‐Aza‐2′‐deoxycytidine (Sigma‐Aldrich) every 24 hours for 3 days.

### 2.8 Western blot analysis

For western blot analyses, cultured cells were washed 3 times with cold PBS on ice and lysed with Laemmli sample buffer (10% SDS glycerol, 1 mol/L Tris‐Cl, pH 6.8) using a scraper. Then, 20 μg of cellular protein was loaded onto 10% SDS‐PAGE gels and transferred to polyvinylidene difluoride membranes. The membranes were immersed in 5% skim milk or 5% BSA in Tris‐buffered saline containing 0.1% Tween 20 for 1 hour and probed with primary antibodies against TEAD4 (H00007004‐M01, Abnova, 1:1000), MEK1 (#9124, Cell Signaling Technology, 1:1000), phospho‐ERK (#9101, Cell Signaling Technology, 1:1000), ERK (#9102, Cell Signaling Technology, 1:1000), YAP1 (#4912, Cell Signaling Technology, 1:1000), phospho‐YAP (#4911, Cell Signaling Technology 1:1000) or β‐actin (#Ab8227, Abcam, 1:5000) overnight at 4°C. Blots were washed and labeled with horseradish peroxidase (HRP)‐conjugated secondary anti‐mouse (sc‐2005) or anti‐rabbit antibodies (sc‐2004, Santa Cruz, 1:5000). Subsequent visualization was detected with a chemiluminescent HRP substrate (Western PICO‐ECL Kit, #PICO‐250 and Western ECL Femto Kit #FEMTO‐100, LPS Solution) and imaged with an ImageQuant LAS 4000 imager (GE Healthcare). Protein changes measured by western blotting were quantified from 3 independent experiments.

### 2.9 Cell proliferation and xenograft assays

Cells (1 × 10^3^) were seeded on a 96‐well plate, and proliferation was measured every 24 hours for 4 days using an EZ‐Cytox Cell Viability Assay Kit (ITSBIO, Seoul, Korea) and a microplate reader (Molecular Devices, Sunnyvale, CA, USA). For the xenograft assay, nonsilencing control or miR‐1271‐expressing MKN74 cells were subcutaneously injected into nude mice (5 × 10^6^ cells/mouse). Tumor volumes were measured with calipers over 4 weeks and calculated by the formula (width)2 × length/2.

### 2.10 Cell migration assays

Cell migration assays were performed in a 24 well transwell chamber (Corning Costar, Corning, NY, USA) fitted with a polycarbonate membrane (8 mm pore size). Cells were washed twice with serum‐free medium, were resuspended in 100 μL (2 × 10^4^ cells/well) serum‐free medium, and added to the upper chamber. The lower chamber was filled with RPMI‐1640 containing 10% FBS. After 20 hours, migrated cells were fixed for 20 minutes with methanol and stained with crystal violet for 6 hours. The membrane was mounted onto glass slides for viewing. The number of cells in 4 randomly chosen microscopic fields was counted. The value of each point was calculated as the average ± SD from 3 independent experiments performed in triplicate.

### 2.11 Cell invasion assays

For cell invasion assays, Matrigel matrix precoated transwell chambers (BD Biosciences, San Jose, CA, USA) were used. Cells were suspended in serum‐free RPMI medium and plated on the upper compartment of the Matrigel matrix. The lower compartment was filled with RPMI medium supplemented with 10% FBS, allowing cells to invade the matrix. After 24 hours, invaded cells were fixed with methanol and stained with crystal violet. The number of cells in 4 randomly chosen microscopic fields was counted. The value of each point was calculated as the average ± SD from 3 independent experiments performed in triplicate.

### 2.12 Luciferase assay

For the luciferase assay, we used the psiCHECK‐2 vector, which contains both a *Renilla* luciferase gene and an independently transcribed firefly luciferase reporter gene. Firefly luciferase activity was used for normalization to account for variations in transfection efficiency and cell viability.^21^ WT and mutant 3′UTR regions of MAP2K1 and TEAD4 were PCR‐amplified, and each amplicon was cloned into the psiCHEK‐2 vector (Promega, Madison, WI, USA). Then, the luciferase assay was performed according to a previously described method.^22^ Briefly, GC cells (1 × 10^5^) plated on a 24‐well tissue culture plate were transfected with 0.5 μg of vector containing the 3′UTR region using Lipofectamine 2000 (Invitrogen). After 24 hours, luciferase activity was measured using a Dual Luciferase Reporter Assay System (Promega) and a Victor plate reader (Perkin Elmer, Waltham, MA, USA). The PCR primer sequences used for cloning are listed in Table S2.

### 2.13 Immunohistochemistry

Paraffin sections of GC tissue were deparaffinized with xylene and then rehydrated. Antigenic retrieval was processed by submerging in citrate buffer (pH 6.0) and microwaving. The sections were then treated with 3% hydrogen peroxide in methanol to quench endogenous peroxidase activity, followed by incubation with 1% bovine serum albumin to block nonspecific binding. The primary anti‐MEK1 (#9124, Cell Signaling Technology, 1:300) antibody was incubated for 1 hour at room temperature. After washing, sections were incubated sequentially with peroxidase‐conjugated secondary antibody and visualized with ChemMate EnVision detection kit (Dako). Slides were manually analyzed by a certified pathologist in a blinded fashion.

### 2.14 Statistical analysis

All data are representative of at least 3 separate experiments, and the results are expressed as the group means ± standard deviations. For two‐group comparisons, Mann‐Whitney tests were performed using R software. *P* < .05 was considered statistically significant.

### 2.15 Data access

All sequencing data, including MBD‐seq, miRNA‐seq, and RNA‐seq data, are available at NCBI SRA (https://www.ncbi.nlm.nih.gov/geo/) via accession numbers GSE46595 and GSE87785.

## 3 RESULTS

### 3.1 Methylated and down‐regulated miRNAs in EpCAM+/CD44+ GC cells

**Figure 1:**
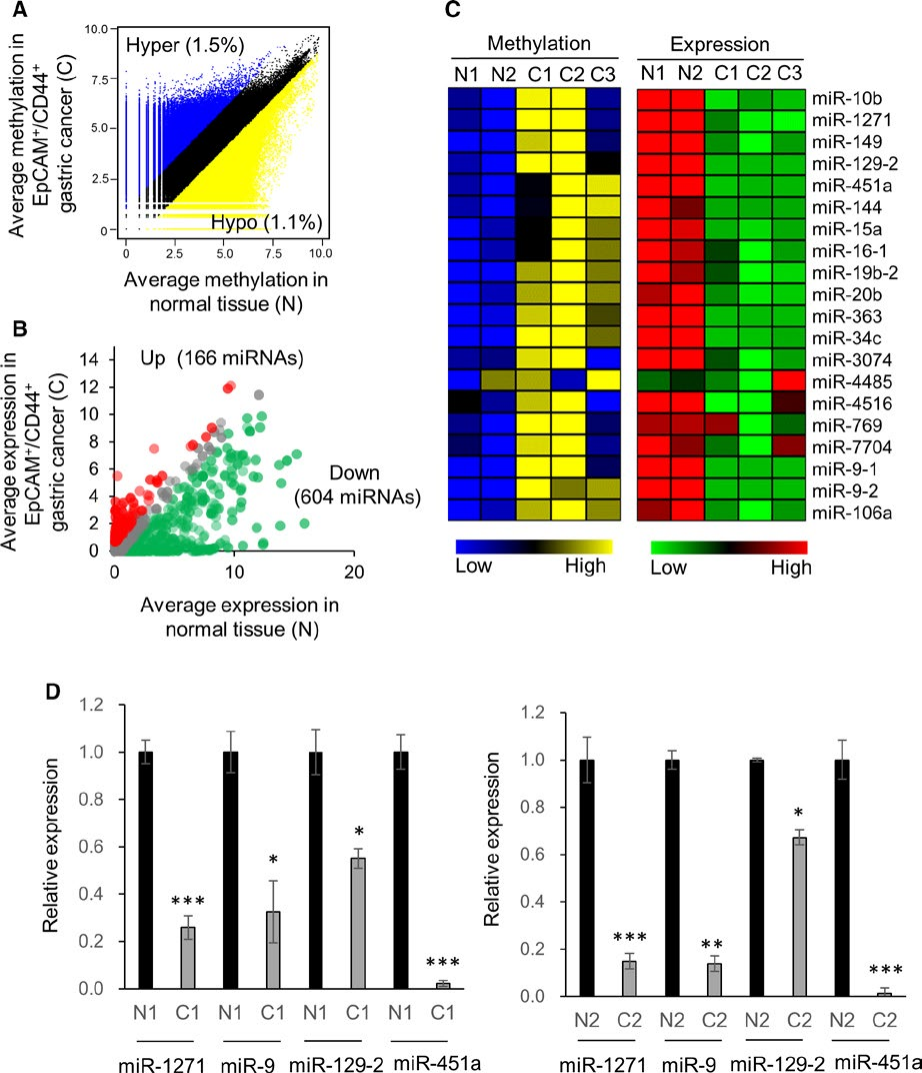
Hypermethylated and down‐regulated miRNAs in EpCAM+/CD44+ GC cells. A, DNA methylation values of EpCAM+/CD44+ GCs vs normal tissues in the human genome are plotted. Blue dots indicate a twofold increase, and yellow dots indicate a twofold decrease. In total, all data were subjected to 100‐bp binning, and the criterion was set as the absolute value of the log_2_ fold change. B, Expression levels of miRNAs in EpCAM+/CD44+ GCs vs normal tissues are plotted. Red dots indicate a twofold increase, and green dots indicate a twofold decrease. C, Combined DNA methylation and gene expression of the top 20 hypermethylated miRNAs are shown as heatmaps. N1 and N2, normal tissues; C1, C2, and C3, 3 EpCAM+/CD44+ GC cell populations. D, Expression levels of selected miRNAs in N1, C1, N2, and C2. qRT‐PCR was performed in triplicate. The mean ± SD is shown, and P‐values were determined using Mann‐Whitney tests. *P <* *.05, **P < .01, ***P < .001

To identify tumor‐suppressive miRNAs repressed by DNA methylation in GC, we performed miRNA‐seq and MBD‐seq on EpCAM+/CD44+ cell populations isolated from 3 primary GC tissues and 2 adjacent normal tissues.^10^ We used EpCAM+/CD44+ GC cells for the following reasons: (1) Since primary tumors are highly heterogeneous, selection of a specific cell population may suppress confounding effects driven by low tumor purity, and (2) GC cells positive for EpCAM or CD44 are highly tumorigenic.^23^ We identified 427 022 (~1.5% of the whole genome) regions with hypermethylation and 317 138 (~1.1%) regions with hypomethylation in GC by MBD‐seq analysis (fold change > 2; Figure 1A), revealing 122 differentially methylated miRNAs in GC. Among the miRNAs examined by miRNA‐seq analysis (n = 1870), 166 were up‐regulated and 604 were down‐regulated in GC (fold change > 2; Figure 1B). The top 20 hypermethylated miRNAs selected based on fold differences in methylation levels and their levels of expression are shown in Figure 1C. To validate the expression differences of these differentially methylated miRNAs, we performed qRT‐PCR analysis of 4 candidate miRNAs (miR‐1271, miR‐9, miR‐129‐2, and miR‐451a), revealing that their levels of expression were concurrently diminished in GC (Figure 1D).

### 3.2 miR‐1271 is hypermethylated and down‐regulated in GC

We also found miR‐1271 to be one of the top 20 hypermethylated miRNAs in EpCAM+/CD44+ GC cells (Figure 1C). Several recent studies demonstrated that miR‐1271 acts as a tumor suppressor across multiple cancer types, including GC.^24^, ^25^, ^26^, ^27^, ^28^ However, because the mechanisms underlying the regulation of miR‐1271 expression are largely unknown, we examined whether DNA hypermethylation is a major contributor to the regulation of miR‐1271 expression.

**Figure 2:**
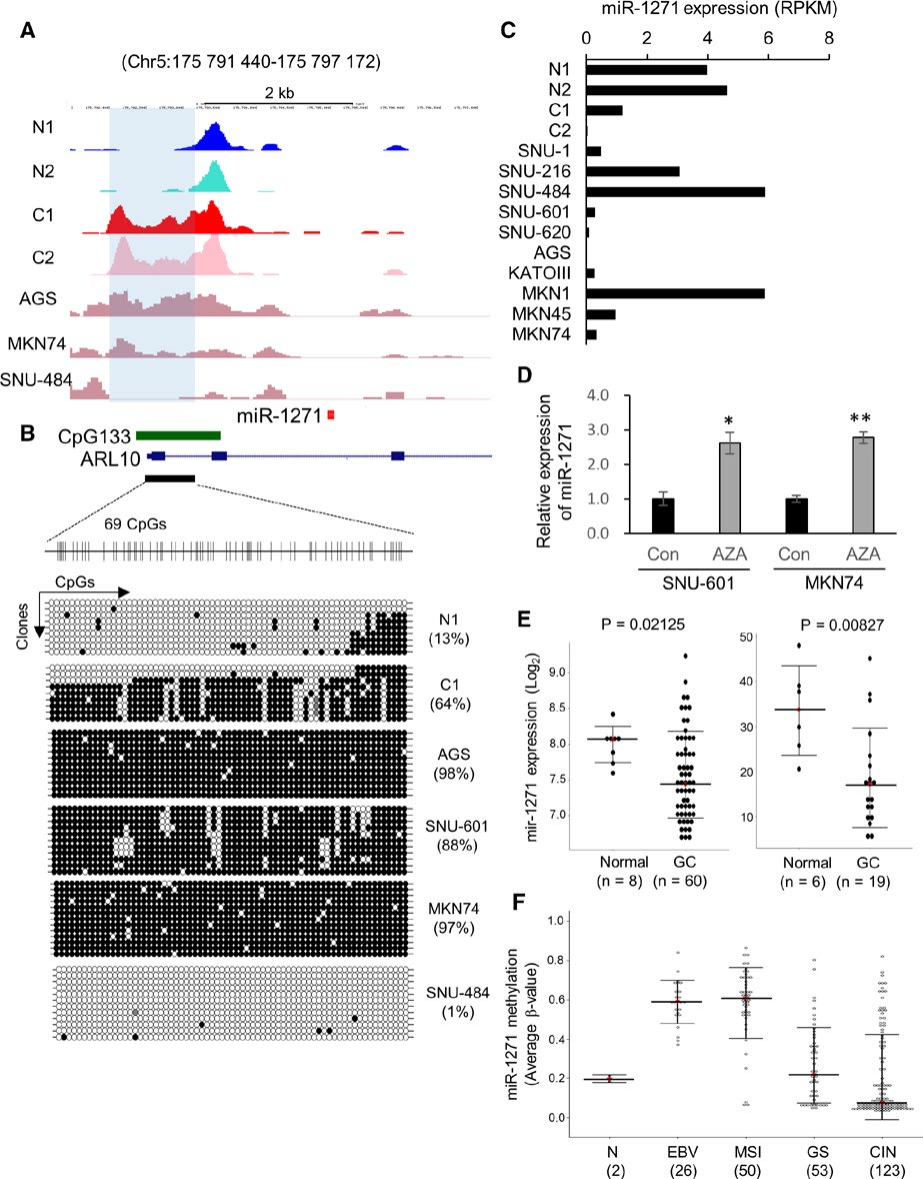
miR‐1271 is repressed by DNA hypermethylation in GC. A, DNA methylation patterns around miR‐1271 in normal tissues (N1 and N2), EpCAM+/CD44+ GC cells (C1 and C2), and GC cell lines (AGS, MKN74, and SNU‐484) as determined by MBD‐seq analyses. B, Bisulfite sequencing for 69 CpG sites within CpG133. Filled and open circles indicate methylated and unmethylated CpGs, respectively. C, Expression level of miR‐1271 in normal tissues (N1 and N2), EpCAM+/CD44+ GC cells (C1 and C2), and GC cell lines as determined by miRNA‐seq. RPKM, reads per kilobase million. D, Restoration of miR‐1271 expression in SNU‐601 and MKN74 cells upon 5‐Aza‐2′‐deoxycytidine (AZA) treatment. The mean ± SD is shown (n = 3), and *P*‐values were determined using Mann‐Whitney tests. *P < .05, **P < .01. E, Reduced expression of miR‐1271 in GC tumor masses compared to that in normal tissues from public datasets (GSE26595 (left) and GSE36968 (right)). Data in the graphs are presented as the mean ± SD, and *P*‐values were determined using Mann‐Whitney tests. F, Methylation level of miR‐1271 in GC subtypes. Methylation array data from 252 GC patients from TCGA are plotted. Data in the graphs are presented as the mean ± SD. EBV, Epstein‐Barr virus; MSI, microsatellite instability; GS, genomically stable; CIN, chromosomal instability

MBD‐seq analysis revealed that the methylation pattern in the miR‐1271 vicinity, located approximately 2 kb upstream from miR‐1271 and overlapped with a CpG island (CpG133), is highly variable in several GC cell types (Figure 2A). Two normal gastric tissues (N1 and N2) and SNU‐484 cells exhibited a methylation‐free state in this region, whereas other GC cell types, AGS, MKN74, and 2 EpCAM+/CD44+ GC cell populations (C1 and C2), showed hypermethylation (Figure 2A). Consistent with the MBD‐seq results, bisulfite sequencing confirmed the methylation profile (Figure 2B). Moreover, the expression level of miR‐1271 estimated by miRNA‐seq tended to be inversely correlated with the methylation pattern (Figure 2C). Additionally, treatment with a demethylating agent, 5‐Aza‐2′‐deoxycytidine**,** recovered miR‐1271 expression approximately 2.6‐fold relative to the basal level in SNU‐601 and MKN74 cells (Figure 2D). Public datasets consistently showed a tendency toward reduced miR‐1271 expression in GC tumor masses compared to that in normal tissues (Figure 2E). To validate the DNA methylation of miR‐1271, we surveyed clinical data encompassing 252 GC patients by downloading methylation array data from The Cancer Genome Atlas (TCGA). Among these TCGA GC patients, those classified as having the Epstein‐Barr virus (EBV) or microsatellite instability (MSI) subtypes^1^ had significantly higher DNA methylation levels around miR‐1271 compared to those of the genomically stable (GS) and chromosomal instability (CIN) subtypes (Figure 2F). Taken together, these results indicate that miR‐1271 is frequently repressed by DNA hypermethylation in GC.

### 3.3 Tumor suppressive role of miR‐1271 in GC

**Figure 3:**
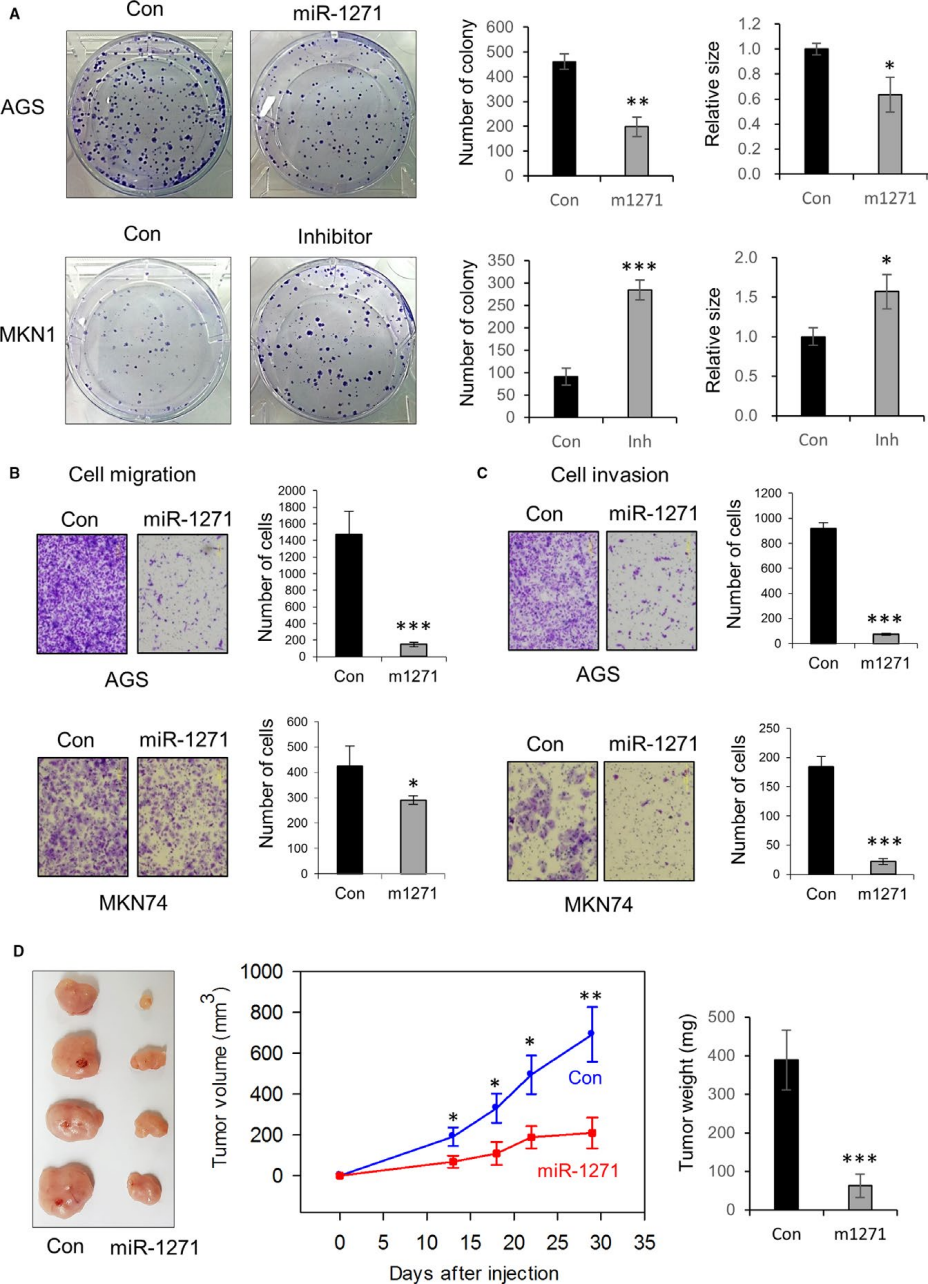
miR‐1271 has a tumor‐suppressive role in GC. A, Clonogenicity of AGS cells upon forced miR‐1271 expression (upper panel) and MKN1 cells upon forced expression of a miR‐1271 inhibitor (lower panel). The numbers and relative sizes of the colonies were measured by comparing nonsilencing control (Con) and miR‐1271 (m1271)‐ or miR‐1271 inhibitor (Inh)‐expressing cells. The graphs represent 3 independent experiments performed in triplicate. Mean ± SD (n = 3). Mann‐Whitney test. *P < .05, **P < .01, ***P < .001. B, C, Migration (B) and invasion (C) of GC cells transfected with a nonsilencing control or a miR‐1271 mimic. The numbers of cells were counted, and representative images are shown. The graphs represent 3 independent experiments performed in triplicate. Mean ± SD (n = 3). Mann‐Whitney test. *P < .05, ***P < .001. D, Tumor volumes and weights were measured after the injection of nonsilencing control cells or miR‐1271‐expressing MKN74 cells into nude mice (n = 4 per group). Mean ± SD. Mann‐Whitney test. *P < .05, **P < .01, ***P < .001

Because the EBV and MSI subtypes have an extensive global hypermethylation phenotype, referred to as EBV‐CIMP (CpG island methylator phenotype) and MSI‐CIMP,^4^ respectively, determining whether the hypermethylation‐mediated down‐regulation of miR‐1271 drives the development of GC is necessary. To assess the functional role of miR‐1271 in GC, we established stable cell lines expressing miR‐1271 using AGS and MKN74 cells (low miR‐1271 expression) or a miR‐1271 inhibitor using MKN1 cells (high miR‐1271 expression). Forced expression of miR‐1271 substantially decreased the clonogenic ability of AGS cells, resulting in reduced colony counts and sizes (Figure 3A). In contrast, inhibition of endogenously expressed miR‐1271 led to the enhanced clonogenicity of MKN1 cells, resulting in a significant increase in colony number and size (Figure 3A). Additionally to cell growth, the migration and invasion capabilities were greatly decreased upon transfection of the miR‐1271 mimic into AGS and MKN74 cells (Figure 3B,C). Notably, when MKN74 cells forced to express miR‐1271 were injected into nude mice, the tumor volumes and weights were significantly decreased in vivo (Figure 3D). Accordingly, these results suggest that miR‐1271 has a tumor suppressive role in GC.

### 3.4 miR‐1271 regulates cancer‐associated genes in GC

Given that the biological role of miRNAs involves repressing the expression of multiple target genes, a comprehensive identification of target genes may help understand the functional roles of miRNAs. We performed RNA‐seq analysis to identify miR‐1271 target genes. For this analysis, we transfected 2 GC cell lines (SNU‐601 and MKN74) with a nonsilencing control or a miR‐1271 mimic and compared gene expression profiles. Genes down‐regulated by miR‐1271 (hereafter referred to as the down‐geneset) were selected based on an expression level reduction cutoff of 1.5‐fold (Table S1). To infer the biological function of miR‐1271, we characterized the biological features of the down‐geneset, revealing that they were significantly enriched for cancer‐associated biological terms, including apoptosis, cell proliferation, angiogenesis, cell migration, and cell cycle arrest (Figure S1A). Additionally, the representative cancer signaling pathways, including the MAP kinase (MAPK) pathway and RAS signaling transduction, were also significantly enriched (Figure S1A).

**Figure 4:**
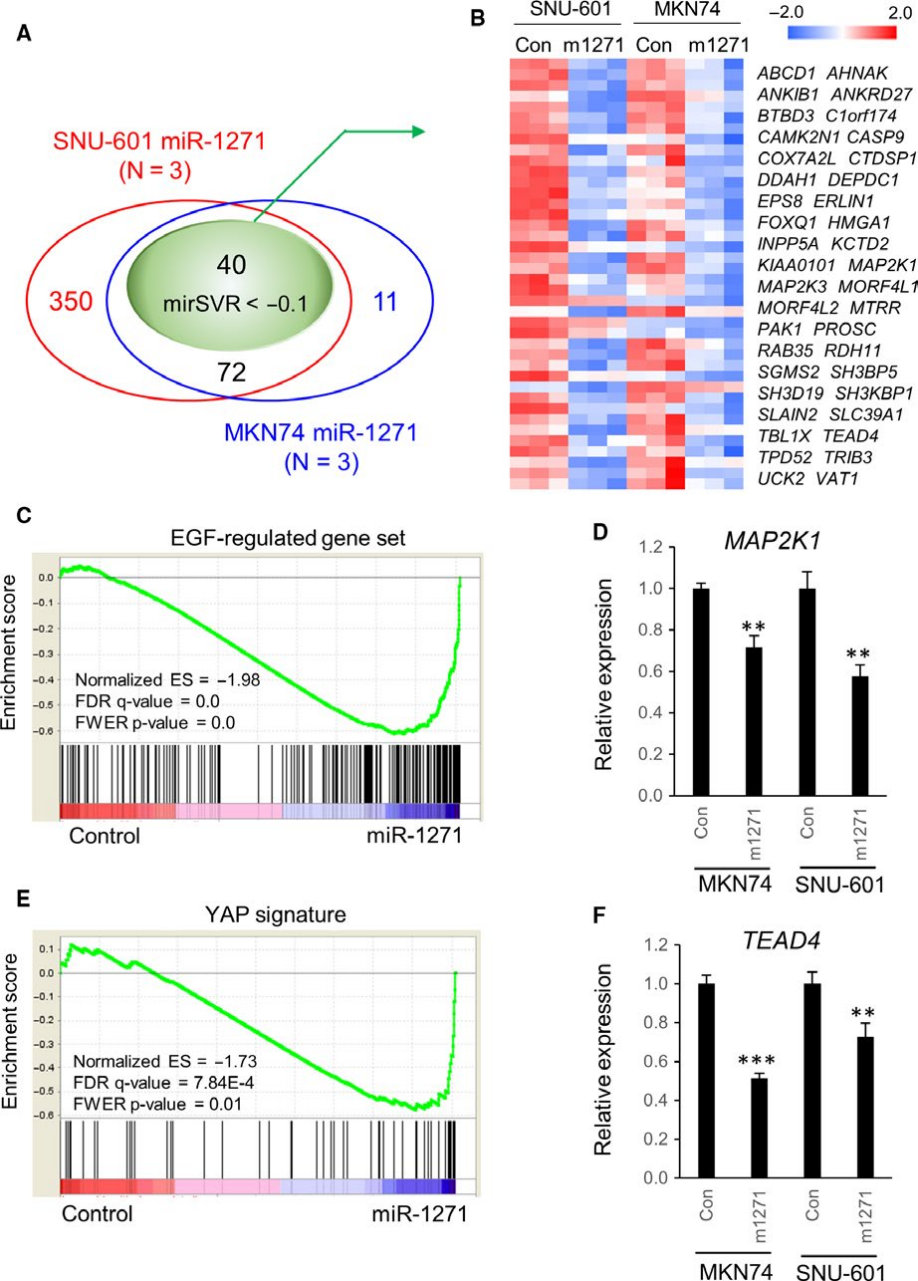
Genes down‐regulated by miR‐1271 are involved in cancer‐associated signaling pathways. A, Overlap between gene sets down‐regulated by the miR‐1271 mimic derived from RNA‐seq analyses of SNU‐601 and MKN74 cells and genes with mirSVR scores less than −0.1 based on conservation (from microRNA.org). Candidate miR‐1271 target genes were narrowed to 40 genes. B, Expression levels of 40 candidate miR‐1271 target genes shown as heatmaps. C, RNA‐seq data were subjected to GSEA analysis (using c6.all.v5.2.symbols.gmt from MSigDB), revealing significant enrichment of the “EGF‐regulated gene set” in a gene set down‐regulated by miR‐1271. D, Expression levels of *MAP2K1* upon transfection of a nonsilencing control (Con) or a miR‐1271 mimic (m1271) into MKN74 and SNU‐601 cells. Mean ± SD (n = 3). Mann‐Whitney test. **P < .01. E, GSEA analysis reveals significant enrichment of the “YAP signature” in a gene set down‐regulated by miR‐1271. F, Expression levels of *TEAD4* upon transfection of a nonsilencing control or a miR‐1271 mimic into MKN74 and SNU‐601 cells. Mean ± SD (n = 3). Mann‐Whitney test. **P < .01, ***P < .001

Next, we examined the overlap between miR‐1271‐regulated genes and putative miR‐1271 target genes that were predicted based on their mirSVR scores^29^ and conservation (from microRNA.org). To identify candidate miR‐1271 target genes, we integrated the down‐genesets derived from the RNA‐seq analysis of SNU‐601 and MKN74 cells, revealing a substantial intersection (n = 112) between the 2 separate down‐genesets (Figure 4A). Further selection from the 112 overlapped genes based on mirSVR scores less than ‐0.1 narrowed the list to the final 40 genes (Figure 4B).

To explore the cancer signaling pathways associated with the final 40 genes, we conducted gene set enrichment analysis (GSEA) using RNA‐seq data, revealing the “EGF‐regulated gene set” as one of the most significantly enriched biological processes within the miR‐1271 down‐geneset (Figure 4C). The final 40 genes included *MAP2K1*, which encodes MEK1, an essential kinase of the EGFR‐Ras‐Raf‐MEK‐ERK signaling cascade. In support of this result, gene sets upregulated by EGFR, KRAS, RAF, and MEK1 activation were concurrently enriched within the miR‐1271 down‐geneset (Figure S1B). The EGFR‐Ras‐Raf‐MEK‐ERK signaling pathway promotes cell proliferation, survival, and metastasis and is aberrantly activated by somatic mutations and gene amplifications in various cancers.^30^ Therefore, regulation of *MAP2K1* expression by miR‐1271 may be important to GC development. To validate whether *MAP2K1* expression is repressed by miR‐1271*,* we measured the relative expression of *MAP2K1* upon miR‐1271 overexpression using qRT‐PCR. Consistent with the RNA‐seq analysis, transfection of a miR‐1271 mimic into MKN74 and SNU‐601 cells resulted in decreased *MAP2K1* expression (Figure 4D).

GSEA also showed that the “YAP signature” was significantly enriched within the miR‐1271 down‐geneset (Figure 4E). YAP1 is an oncogenic transcriptional coactivator that is inhibited by phosphorylation through the Hippo signaling pathway.^31^ *TEAD4*, an oncogenic transcription factor that interacts with YAP1 in GC, was also among the final 40 genes (Figure 4B).^18^ Due to the link between TEAD4 and YAP1, we suspected that repression of *TEAD4* by miR‐1271 may regulate the “YAP signature”. Indeed, forced expression of a miR‐1271 mimic markedly decreased *TEAD4* expression in both MKN74 and SNU‐601 cells (Figure 4F).

### 3.5 miR‐1271 enhances the ERK/MAPK signaling pathway by targeting MEK1 in GC

**Figure 5:**
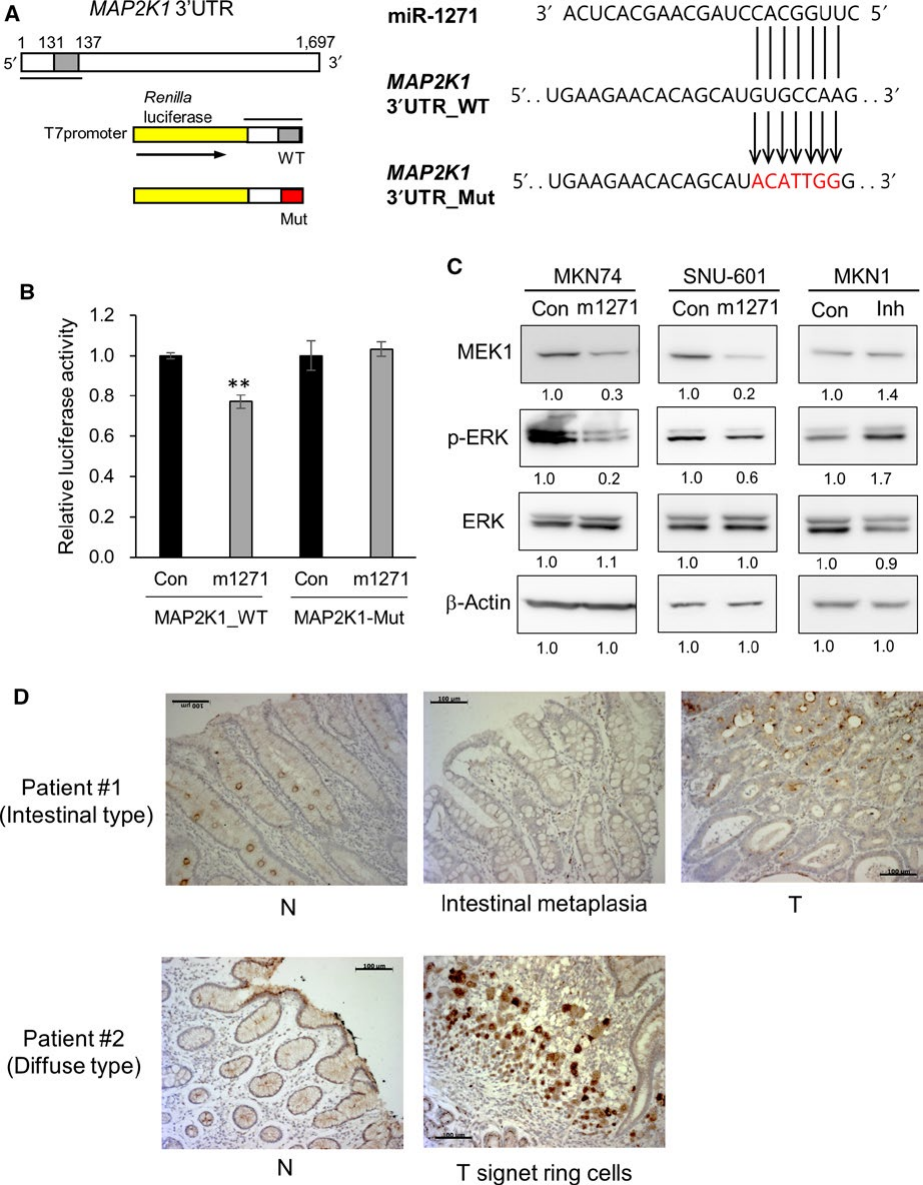
MEK1 is a direct target of miR‐1271. A, *MAP2K1* has one potential miR‐1271 complementary binding site within its 3′UTR (position 131‐137). The 3′UTR (positions 1‐138) of *MAP2K1* was cloned into a reporter vector (psiCHECK2‐MAP2K1‐WT or psiCHECK2‐MAP2K1‐Mut) downstream of the *Renilla* luciferase gene between *Pme*I and *Xho*I. A schematic representation of the miR‐1271 seed region in the *MAP2K1* 3′UTR (*MAP2K1* 3′UTR‐WT) and the mutated 3′UTR (*MAP2K1* 3′UTR‐Mut) is shown on the right. B, Cotransfection of MKN74 cells with a miR‐1271 mimic and psiCHECK2‐MAP2K1‐WT resulted in a significant decrease in luciferase activity. The graphs represent 3 independent experiments performed in triplicate. Mean ± SD (n = 3). Mann‐Whitney test. **P < .01. C, Levels of MEK1, phosphorylated ERK, total ERK, and β‐actin (loading control) upon transfection of a nonsilencing control (Con), a miR‐1271 mimic (m1271), or a mir‐1271 inhibitor (Inh). D, Immunohistochemistry analysis of MEK1 in tissue sections from GC patients. Paraffin‐embedded sections of matched normal samples and tumor samples were examined for MEK1 expression using an anti‐MEK1 antibody (1:300 dilution). Tumor tissue showed higher MEK1 expression than normal or intestinal metaplasia tissues in intestinal‐type GC (Patient #1). High MEK1 expression was detected in signet ring cells in diffuse‐type GC (Patient #2). Scale bars, 100 μm

*MAP2K1* mRNA has a potential miR‐1271 complementary binding site within its 3′UTR (positions 131‐137, mirSVR score: −0.8255). To validate whether *MAP2K1* (MEK1) is a direct target of miR‐1271, we cloned the *MAP2K1* 3′UTR (positions 1‐138) into a reporter vector (psiCHECK2‐MAP2K1‐WT or psiCHECK2‐MAP2K1‐Mut) downstream of the *Renilla* luciferase gene between *Pme*I and *Xho*I (Figure 5A). Cotransfection of the miR‐1271 mimic with psiCHECK2‐MAP2K1‐WT significantly prohibited luciferase activity (Figure 5B), while there was no significant difference in luciferase activity between controls and cells cotransfected with the miR‐1271 mimic and the mutated construct.

As expected, the protein expression of MEK1 was also reduced in MKN74 and SNU‐601 cells after transfection of the miR‐1271 mimic (Figure 5C). Because reduction of MEK1 expression may subsequently diminish the activity of the EGFR‐Ras‐Raf‐MEK‐ERK signaling pathway, we measured the level of phosphorylated ERK upon modulation of miR‐1271 expression. Transfecting a miR‐1271 mimic into MKN74 and SNU‐601 cells resulted in a reduction in the level of phosphorylated ERK (Figure 5C), whereas transfection of the miR‐1271 inhibitor into MKN1 cells increased the levels of MEK1 and phosphorylated ERK (Figure 5C). These data indicate that miR‐1271 has an inhibitory role against the ERK/MAPK pathway by repressing MEK1.

To examine the protein expression of MEK1 in GC specimens, we performed immunohistochemistry analysis of 3 sets of tissue sections (2 intestinal‐type GC patients and one diffuse‐type GC patient). MEK1 was detected in the cytoplasms of both normal and tumor tissues (Figure 5D). In the intestinal type, MEK1 was expressed at higher levels in tumor tissue than in normal or intestinal metaplasia tissues. Moreover, high MEK1 expression was observed in signet ring cells in the diffuse type, suggesting that enhanced MEK1 expression has an important role in GC development.

### 3.6 miR‐1271 enhances the YAP signature by directly targeting TEAD4 in GC

**Figure 6:**
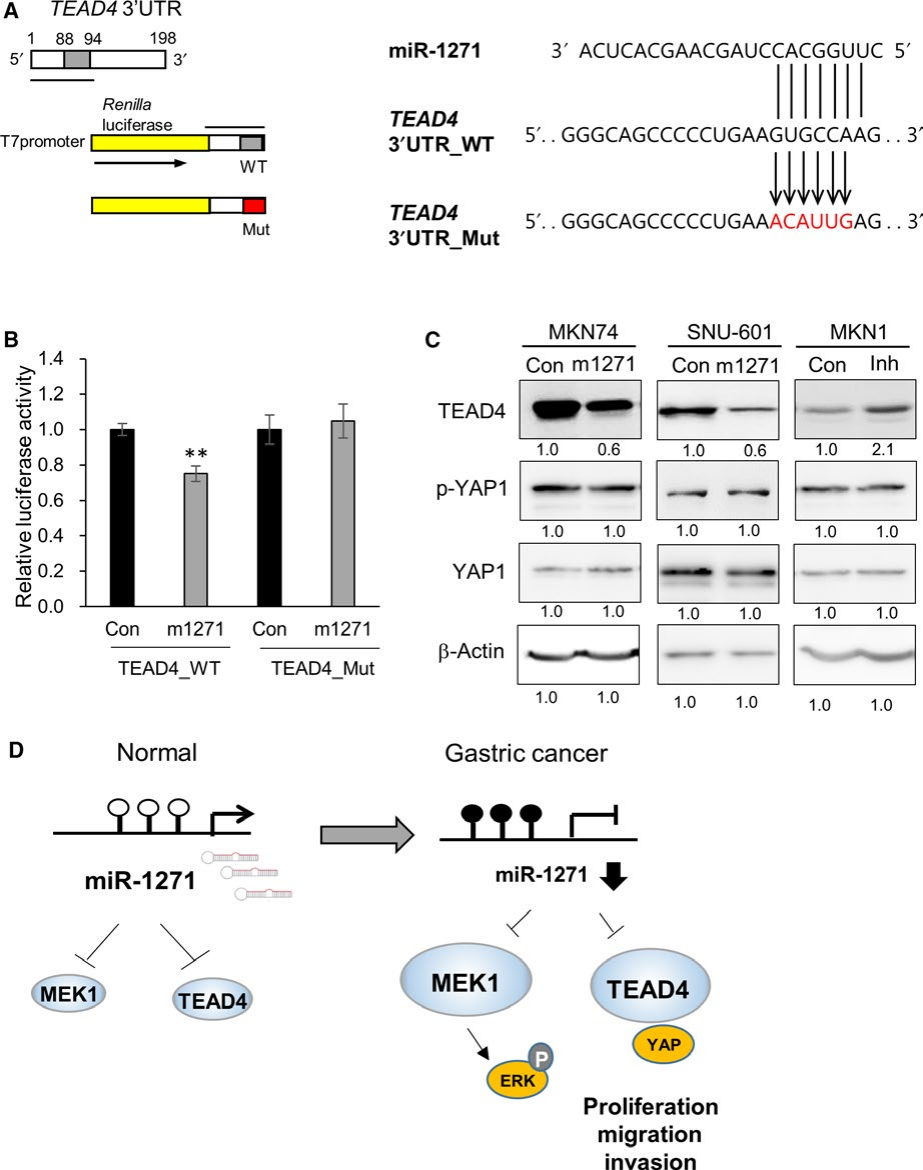
TEAD4 is a direct target of miR‐1271. A, *TEAD4* has one potential miR‐1271 complementary binding site within its 3′UTR (positions 88‐94). The 3′UTR (position 1‐95) of *TEAD4* was cloned into a reporter vector (psiCHECK2‐TEAD4‐WT or psiCHECK2‐TEAD4‐Mut) downstream of the *Renilla* luciferase gene between *Pme*I and *Xho*I. A schematic representation of the miR‐1271 seed region in the *TEAD4* 3′UTR (*TEAD4* 3′UTR‐WT) and the mutated 3′UTR (*TEAD4* 3′UTR‐Mut) is shown on the right. B, The luciferase activity of MKN74 cells cotransfected with a miR‐1271 mimic and psiCHECK2‐TEAD4‐WT was significantly decreased. The graphs represent 3 independent experiments performed in triplicate. Mean ± SD (n = 3). Mann‐Whitney test. **P < .01. C, Levels of TEAD4, phosphorylated YAP1, total YAP1, and β‐actin (loading control) upon transfection with a nonsilencing control (Con), a miR‐1271 mimic (mimic), or a miR‐1271 inhibitor (Inh). D, Model of miR‐1271 silencing inducing GC cell proliferation, migration, and invasion via the enhanced expression of MEK1 and TEAD4. In GC, DNA methylation represses miR‐1271 expression, which activates the ERK/MAPK signaling pathway and the YAP signature by up‐regulating MEK1 and TEAD4

*TEAD4* mRNA contains a potential miR‐1271 complementary binding site within its 3′UTR (positions 88‐94, mirSVR score: −0.5184). To validate whether TEAD is a direct target of miR‐1271, we cloned the 3′UTR (positions 1‐95) of *TEAD4* into a reporter vector (psiCHECK2‐TEAD4‐WT or psiCHECK2‐TEAD4‐Mut; Figure 6A). Cotransfection of the miR‐1271 mimic with psiCHECK2‐TEAD4‐WT resulted in a significant reduction in luciferase activity (Figure 6B), while cotransfection of the mutated construct with the miR‐1271 mimic had no significant effect on luciferase activity.

We next examined whether TEAD4 protein expression is also controlled by miR‐1271. The miR‐1271 mimic reduced the level of TEAD4 protein expression in MKN74 and SNU‐601 cells, whereas the miR‐1271 inhibitor induced up‐regulation of the TEAD4 protein in MKN1 cells (Figure 6C). However, the level of total and phosphorylated YAP1 was not changed by miR‐1271. These data indicate that enrichment of the “YAP signature” within the down‐geneset may be partially attributed to the down‐regulation of TEAD4 but not to the regulation of YAP1 activity.

## 4 DISCUSSION

miRNAs play crucial roles in normal development and disease pathogenesis through posttranscriptional gene regulation,^32^ and an expanding body of literature supports that expression of miRNAs themselves can be controlled by epigenetic mechanisms, such as DNA methylation and histone modifications. In this study, we identified tumor‐suppressive miRNAs repressed by DNA methylation in GC. Using high‐throughput sequencing of EpCAM+/CD44+ GC cells, we identified a set of miRNAs that are hypermethylated and down‐regulated in GC. Consistent with previous studies,^33^, ^34^, ^35^ CpG sites near miR‐129‐2 were hypermethylated and down‐regulated in GC, and miR‐149 was hypermethylated and down‐regulated in GC as well as in colorectal and cervical cancer, as previously reported.^36^, ^37^ Additionally, the tumor suppressors miR‐34c and miR‐9‐1, silenced by DNA hypermethylation in GC,^33^, ^38^ were also hypermethylated and down‐regulated in EpCAM+/CD44+ GC cells. Furthermore, tumor‐suppressive miRNAs, including miR‐451a,^39^ miR‐15a,^40^ and miR‐363,^41^ were hypermethylated and down‐regulated in EpCAM+/CD44+ GC cells.

Among the hypermethylated and down‐regulated miRNAs, we focused on miR‐1271, whose function had not yet been reported at the time of our identification. Ectopic expression of miR‐1271 in GC cells showed a tumor suppressive function in vitro and in vivo. Recently, several targets of miR‐1271 (ZEB1, TWIST1, CCNG1, FOXQ1, ALK, HOXA5, GPC3, IGF1R, IRS1, mTOR, and BCL2) were identified in various cancers using a knowledge‐based approach.^24^, ^25^, ^26^, ^27^, ^42^, ^43^ However, since genome‐wide screening has not yet been done, we performed RNA‐seq analysis to identify miR‐1271 target genes, and GSEA reflected the functional characteristics of the final 40 miR‐1271 targets. Consistent with the functional role of MEK1 as a key signaling molecule of the EGFR‐Ras‐Raf‐MEK‐ERK cascade, the “EGFR‐regulated gene set” and “KRAS signature” were significantly enriched within the genes down‐regulated by miR‐1271 (Figure S1B). In support of this finding, we observed that miR‐1271 repressed MEK1 expression and inhibited the ERK/MAPK signaling pathway. Given that activation of the ERK/MAPK signaling pathway enhances cancer cell growth and survival, the tumor‐suppressive effect of miR‐1271 may be partially mediated by repression of MEK1 (Figure 6D). Furthermore, we found MEK1 to be highly expressed in signet ring cells using immunohistochemistry analysis. Signet ring cell carcinoma appears to be relatively frequent in women and young patients and has a worse prognosis than other forms of GC.^44^, ^45^ However, the molecular basis of signet ring cell growth, differentiation, and metastasis remains unclear. We suggest that the epigenetic silencing of miR‐1271 and enhanced expression of MEK1 may promote the growth and metastasis of signet ring cells. Additional studies are necessary to determine whether the DNA methylation of miR‐1271 is frequent in signet ring cells.

RNA‐seq analysis also revealed the “YAP signature” to be highly enriched within the miR‐1271 down‐geneset. miR‐1271 overexpression resulted in down‐regulation of TEAD4, which contributes to the “YAP signature” as a YAP1‐interacting transcription factor. We previously showed that TEAD4 has oncogenic potential in GC via the transcriptional regulation of cancer‐associated target genes.^18^ Thus, the reduced expression of oncogenic TEAD4 by miR‐1271 may partially contribute to the tumor‐suppressive effect of miR‐1271 in GC (Figure 6D).

Additionally to MEK1 and TEAD4, GSEA analysis also revealed other cancer‐associated gene sets as the significantly enriched signature in the miR‐1271 down‐geneset (Figure S1C). Although these signatures were excluded from this study, these may be important because TGFβ and LEF1 signatures are involved in epithelial‐to‐mesenchymal transition (EMT) and NFκB and RELA signatures are involved in tumor immunity (Figure S1C). Therefore, it is required to examine whether miR‐1271 is involved in the regulation of EMT and tumor immunity.

Treatment with a DNA methylation inhibitor, 5‐Aza‐2′‐deoxycytidine, recovered miR‐1271 expression in SNU‐601 and MKN74 cells, and we further examined whether this drug represses the miR‐1271 targets MEK1 and TEAD4. 5‐Aza‐2′‐deoxycytidine could repress MEK1 in SNU‐601 cells and TEAD4 in MKN74 cells only slightly (Figure S2), suggesting that the epigenetic silencing of miR‐1271 may be only a partial mechanism underlying the enhanced expression of MEK1 and TEAD4 in GC. Recent CRISPR‐based epigenomic editing technologies, such as the dCas9‐Tet1 system, may elucidate a more direct effect of miR‐1271 demethylation.^46^

In conclusion, we demonstrated that the tumor‐suppressive role of miR‐1271 is repressed by DNA methylation in GC. This study identified *MAP2K1* and *TEAD4* as miR‐1271 target genes that are involved in ERK/MAPK and YAP1 signaling pathways, respectively. While further studies are required to demonstrate the detailed functions of miR‐1271, this study presents miR‐1271 and its target genes as potential therapeutic targets in GC.

## CONFLICTS OF INTEREST

The authors declare no competing financial interests.
