# Supplementary figures and images for: Epigenetic silencing of miR‐1271 enhances MEK1 and TEAD4 expression in gastric cancer
Source: Cancer Med. 2018 Jun 4;7(7):3411–24. doi: 10.1002/cam4.1605 (PMC6051202; doi:10.1002/cam4.1605)

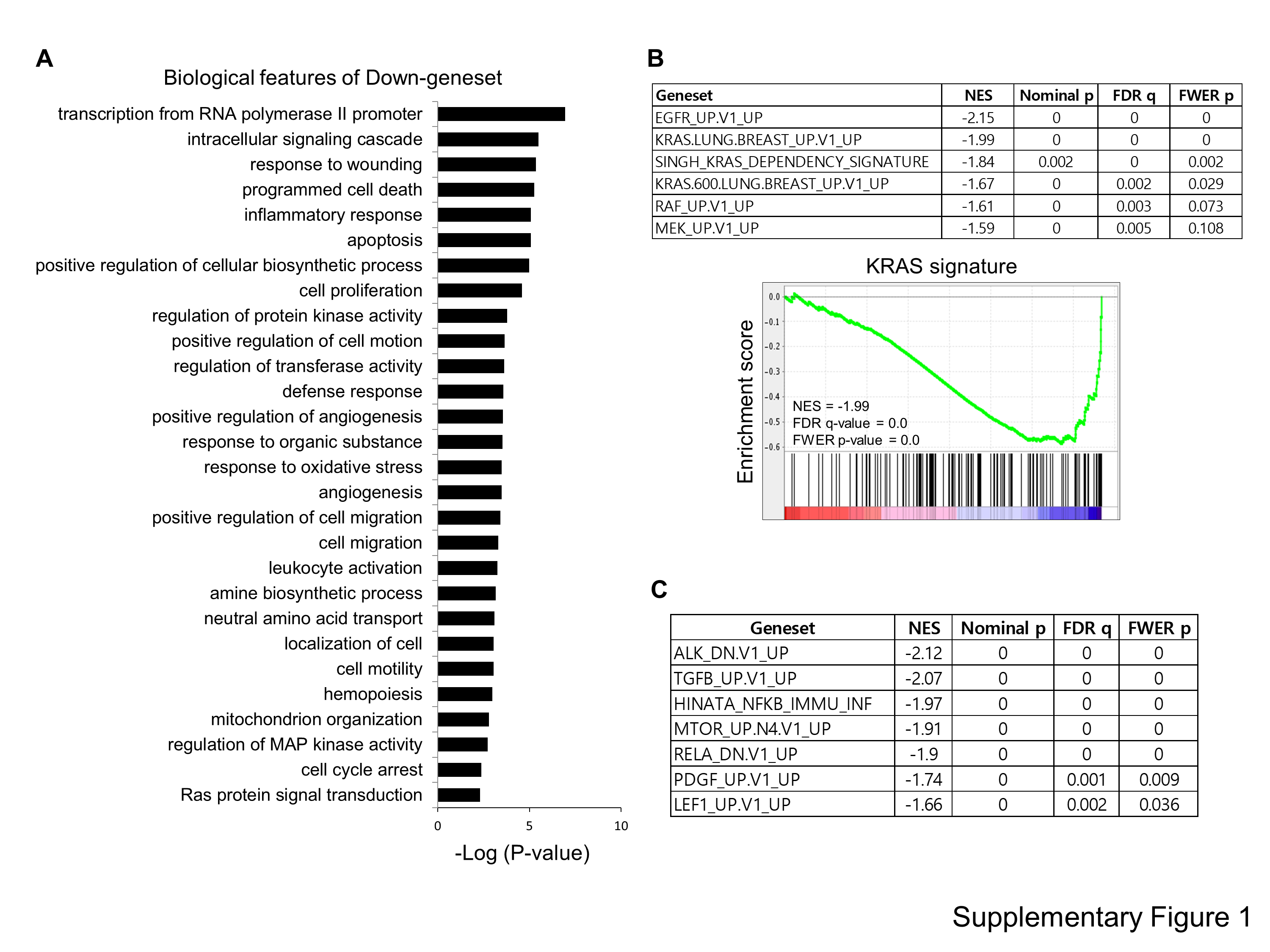

Supplement: Supplementary file 1 [file CAM4-7-3411-s001.tif]

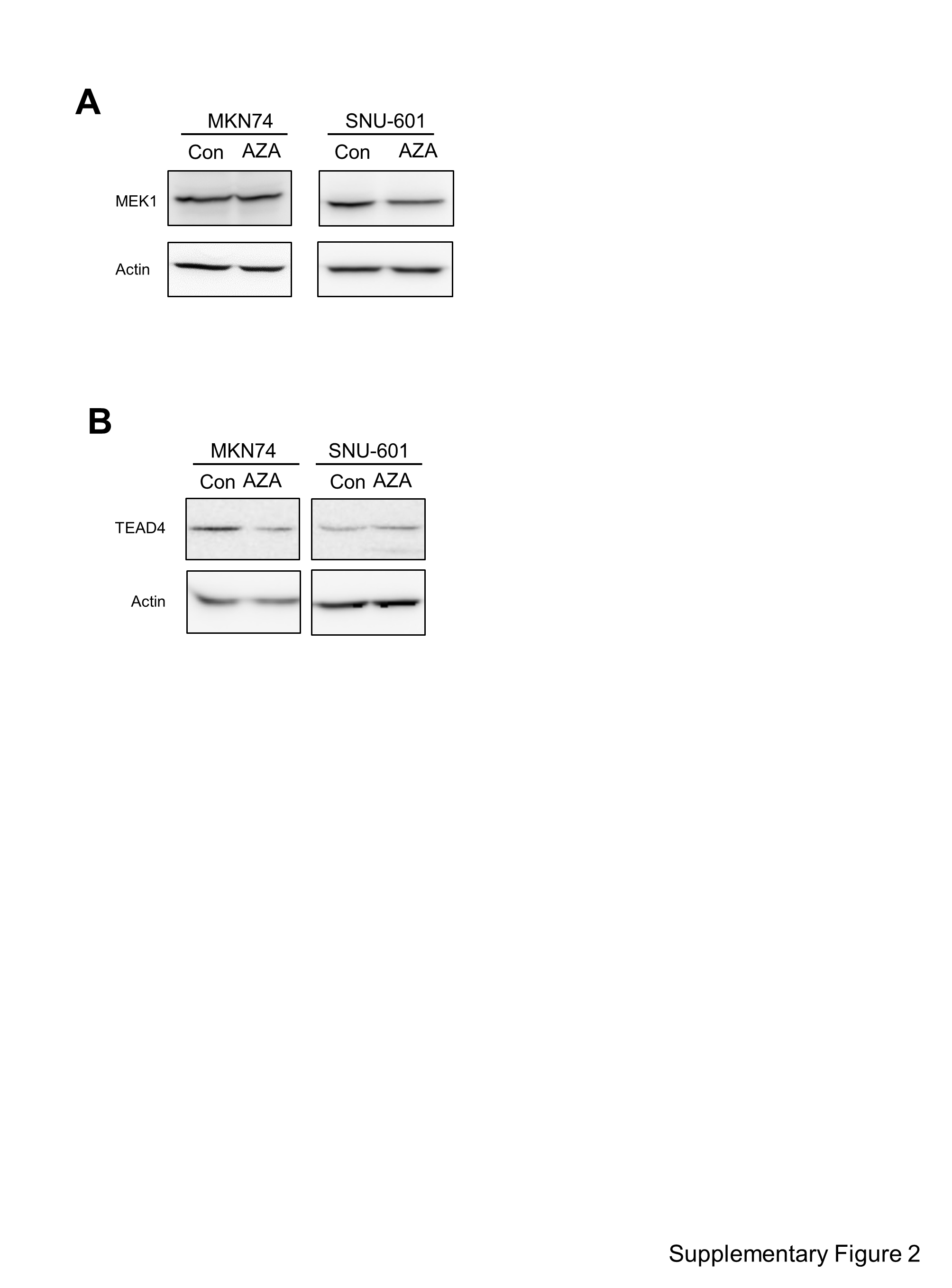

Supplement: Supplementary file 2 [file CAM4-7-3411-s002.tif]
